# Factorization in Call-by-Name and Call-by-Value Calculi via Linear Logic

**DOI:** 10.1007/978-3-030-71995-1_11

**Published:** 2021-03-23

**Authors:** Claudia Faggian, Giulio Guerrieri

**Affiliations:** 8 University of Oxford, Oxford, UK; 9 Sorbonne Université - LIP6, Paris, France; 10 Université de Paris, IRIF, CNRS, F-75013 Paris, France; 11 University of Bath, Department of Computer Science, Bath, UK

## Abstract

In each variant of the $\lambda$-calculus, factorization and normalization are two key properties that show how results are computed. Instead of proving factorization/normalization for the call-by-name (CbN) and call-by-value (CbV) variants separately, we prove them only once, for the bang calculus (an extension of the $\lambda$-calculus inspired by linear logic and subsuming CbN and CbV), and then we transfer the result via translations, obtaining factorization/normalization for CbN and CbV.

The approach is robust: it still holds when extending the calculi with operators and extra rules to model some additional computational features.

### 1 Introduction

The $\lambda$-calculus is the model of computation underlying functional programming languages and proof assistants. Actually there are many $\lambda$-calculi, depending on the *evaluation mechanism* (for instance, call-by-name and call-by-value—CbN and CbV for short) and *computational features* that the calculus aims to model.

In $\lambda$-calculi, a rewriting relation formalizes computational steps in program execution, and normal forms are the results of computations. In each calculus, a key question is to define a *normalizing strategy*: How to compute a result? Is there a reduction strategy which is guaranteed to output a result, if any exists?

Proving that a calculus admits a normalizing strategy is complex, and many techniques have been developed. A well-known method first proves *factorization* [4,32,19,2]. Given a calculus with a rewriting relation $\rightarrow$, a strategy $\underset{\mathrm{L}}{\rightarrow} \subseteq \rightarrow$ *factorizes* if $\rightarrow^* \subseteq \underset{\mathrm{L}}{\rightarrow}^* \cdot \underset{\neg\mathrm{L}}{\rightarrow}^*$ ($\underset{\neg\mathrm{L}}{\rightarrow}$ is the dual of $\underset{\mathrm{L}}{\rightarrow}$), *i.e.* any reduction sequence can be rearranged so as to perform $\underset{\mathrm{L}}{\rightarrow}$-steps first and then the other steps. If, moreover, the strategy satisfies some "good properties", we can conclude that the strategy is normalizing. Factorization is important also because it is commonly used as a building block in the proof of other properties of the *how-to-compute* kind. For instance, *standardization*, which generalizes factorization: every reduction sequences can be rearranged according to a predefined order between redexes.

*Two for One.* CbN and CbV $\lambda$-calculi are two distinct rewriting systems. Quoting from Levy [20]: *the existence of two separate paradigms* (CbN and CbV) is troubling because to prove a certain property—such as factorization or normalization—for both systems *we always need to do it twice.*

The *first aim* of our paper is to develop a technique for deriving factorization for both the CbN [4] and CbV [27] $\lambda$-calculi as corollaries of a *single* factorization theorem, and similarly for normalization. A key tool in our study is the *bang calculus* [11,15], a calculus inspired by linear logic in which CbN and CbV embed.

*The Bang Calculus.* The bang calculus is a variant of the $\lambda$-calculus where an operator ! plays the role of a marker for non-linear management: duplicability and discardability of resources. The bang calculus is nothing but Simpson's linear $\lambda$-calculus [31] without linear abstraction, or the untyped version of the implicative fragment of Levy's Call-by-Push-Value [20], as first observed by Ehrhard [10].

The motivation to study the bang calculus is to have a general framework where both CbN and CbV $\lambda$-calculi can be simulated, via two distinct *translations* inspired by Girard's embeddings [14] of the intuitionistic arrow into linear logic. So, a certain property can be studied in the bang calculus and then automatically transferred to the CbN and CbV settings by translating back.

This approach has so far mainly be exploited semantically [21,10,11,15,9,7], but can be used it also to study operational properties [15,30,13]. In this paper, we push forward this operational direction.

*The Least-Level Strategy.* We study a strategy from the literature of linear logic [8], namely *least-level reduction* $\xrightarrow[\mathrm{L}]{}$, which fires a redex at minimal level—the *level* of a redex is the number of ! under which the redex occurs.

We prove that the least-level reduction factorizes and normalizes in the bang calculus, and then we transfer the same results to CbN and CbV $\lambda$-calculi (for suitable definitions of least-level in CbN and CbV), by exploiting properties of their translations into the bang calculus. A single proof suffices. It is two-for-one! Or even better, three-for-one.

The rewriting study of the least level strategy in the bang calculus is based on simple techniques for factorization and normalization we developed recently with Accattoli [2], which simplify and generalize Takahashi's method [32].

*Subtleties of the Embeddings.* Transferring factorization and normalization results via translation is highly non-trivial, *e.g.* in CPS translations [27]. This applies also to transferring least-level factorization from the bang calculus to the CbN and CbV $\lambda$-calculi. To transfer the property smoothly, the translations should preserve levels and normal forms, which is delicate, in particular for CbV. For instance, the embedding of CbV into the bang calculus defined in [15,30] does not preserve levels and normal forms. As a consequence, the CbV translation studied in [15,30] cannot be used to derive least-level factorization or *any* normalization result in a CbV setting from the corresponding result in the bang calculus.

Here we adopt the refined CbV embedding of Bucciarelli *et al.* [7], which does preserve levels and normal forms. While the preservation of normal forms is already stressed in [7], the preservation of levels is proved here for the first time, and it is based on non-trivial properties of the embedding.

*Beyond pure.* Our *second aim* is to show that the developed technique for the joined factorization and normalization of CbN and CbV via the bang calculus is *robust*. We do so, by studying extensions of all three calculi with operators (or, in general, with extra rules) which model some additional computational features, such as non-deterministic or probabilistic choice. We then show that the technique scales up smoothly, under mild assumptions on the extension.

*A Motivating Example.* Let us illustrate our approach on a simple case, which we will use as a running example. De' Liguoro and Piperno's CbN non-deterministic $\lambda$-calculus $\Lambda_{\oplus}^{\mathsf{cbn}}$ [23] extends the CbN $\lambda$-calculus with an operator $\oplus$ whose reduction $\to_{\oplus}$ models *non-deterministic choice*: $t \oplus s$ rewrites to either $t$ or $s$. It admits a standardization result, from which it follows that the leftmost-outermost reduction strategy (noted $\underset{\mathrm{LO}}{\to}_{\beta\oplus}$) is *complete*: if $t$ has a normal form $u$ then $t \underset{\mathrm{LO}}{\to}_{\beta\oplus}^{*} u$. In [22], de' Liguoro considers also a CbV variant $\Lambda_{\oplus}^{\mathsf{cbv}}$, extending Plotkin CbV $\lambda$-calculus [27] with an operator $\oplus$. One may prove standardization and completeness—again—from scratch, even though the proofs are similar.

The approach we propose here is to work in the bang calculus enriched with the operator $\oplus$. We show that the calculus satisfies *least-level factorization*, from which it follows that the least-level strategy (noted $\underset{\mathrm{L}}{\to}_{\beta_!\oplus}$) is *complete*, *i.e.* if $t$ has a normal form $u$, then $t \underset{\mathrm{L}}{\to}_{\beta_!\oplus}^{*} u$. The translation then guarantees that analogous results hold also in $\Lambda_{\oplus}^{\mathsf{cbn}}$ and $\Lambda_{\oplus}^{\mathsf{cbv}}$, without proving them again.

*The Importance of Being Modular.* The bang calculus with operators is actually a general formalism for several calculi, one calculus for each kind of computational feature modeled by operators. Concretely, the reduction $\to$ consists of $\to_{\beta_!}$ (which subsumes CbN $\to_{\beta}$ and CbV $\to_{\beta_v}$) and other reduction rules $\to_{\rho}$.

We decompose the proof of factorization of $\to$ in modules, by using the *modular approach* we recently introduced together with Accattoli [3].

The key module is the least-level factorization of $\to_{\beta_!}$, because it is where the higher-order comes into play—this is done, once and for all. Then, we consider a generic reduction rule $\to_{\rho}$ to add to $\to_{\beta_!}$. Our general result is that if $\to_{\rho}$ has "good properties" and interacts well with $\to_{\beta_!}$ (which amounts to an easy test, combinatorial in nature), then we have least-level factorization for $\to_{\beta_!} \cup \to_{\rho}$.

Putting all together, when $\to_{\rho}$ is instantiated to a concrete reduction (such as $\to_{\oplus}$), the user of our method only has to verify a simple test (namely Proposition 34), to conclude that $\to_{\beta_!} \cup \to_{\rho}$ has least-level factorization. In particular, factorization for $\to_{\beta_!}$ is a ready-to-use black box the user need not to worry about—our proof is robust enough to hold whatever the other rules are. Finally, the embeddings automatically give least-level factorization for the corresponding CbV and CbN calculi. Section 7 illustrates our method in the case $\to_{\rho} = \to_{\oplus}$.

*Subtleties of the Modular Extensions.* To adopt the modular approach for factorization presented in [3], we have to face an important difficulty that arises when dealing with normalizing strategies, and which is not studied in [3].

A *normalizing* strategy cannot overlook redexes and it usually selects the redex $r$ to fire through a property that $r$ minimizes with respect to the redexes in the whole term, such as being a *least level* redex or being the *leftmost-outermost* (shortened to LO) redex—normalizing strategies are *positional*. The problem is that, in general, if $\to = \to_\beta \cup \to_\rho$, then $\underset{\text{LO}}{\to}$ reduction is not the union of $\underset{\text{LO}}{\to}_\beta$ and $\underset{\text{LO}}{\to}_\rho$: the normalizing strategy of the compound system is not obtained putting together the normalizing strategies of the components. Let us explain the issue on our running example $\to_{\beta\oplus}$, in the familiar case of leftmost-outermost reduction.

*Example 1.* Consider head reductions for $\to_\beta$ and for $\to_{\beta\oplus} = \to_\beta \cup \to_\oplus$, noted $\underset{\mathsf{h}}{\to}_\beta$ and $\underset{\mathsf{h}}{\to}_{\beta\oplus}$, respectively. In the term $s = (\mathsf{II})(x \oplus y)$ where $\mathsf{I} = \lambda x.x$, the subterm $\mathsf{II}$ (a $\beta$-redex) is in head position for both the reduction $\to_\beta$ and its extension $\to_{\beta\oplus}$. So, $s \underset{\mathsf{h}}{\to}_\beta \mathsf{I}(x \oplus y)$ and $s \underset{\mathsf{h}}{\to}_{\beta\oplus} \mathsf{I}(x \oplus y)$. And in the term $t = (x \oplus y)(\mathsf{II})$, the head position is occupied by $x \oplus y$, which is a $\oplus$-redex. Therefore, $\mathsf{II}$ is not the head redex in $t$, neither for $\beta$ nor for $\beta\oplus$. In general, $\underset{\mathsf{h}}{\to}_{\beta\oplus} = \underset{\mathsf{h}}{\to}_\beta \cup \underset{\mathsf{h}}{\to}_\oplus$.

In contrast, for leftmost-outermost reduction $\underset{\text{LO}}{\to}_{\beta\oplus}$, which reduces the LO-redex, we have $\underset{\text{LO}}{\to}_{\beta\oplus} \neq \underset{\text{LO}}{\to}_\beta \cup \underset{\text{LO}}{\to}_\oplus$. Consider again the term $t = (x \oplus y)(\mathsf{II})$. Since $x \oplus y$ is not a $\beta$-redex, $\mathsf{II}$ is the LO-redex for $\to_\beta$. Instead, $\mathsf{II}$ is not the LO-redex for $\to_{\beta\oplus}$ (here the LO-redex is $x \oplus y$). So, $t \underset{\text{LO}}{\to}_\beta (x \oplus y)\mathsf{I}$ but $t \underset{\text{LO}}{\not\to}_{\beta\oplus} (x \oplus y)\mathsf{I}$.

The least-level factorization for $\to_{\beta_!}$, $\to_\beta$, and $\to_{\beta_v}$ we prove here is robust enough to make it ready to be used as a module in a larger proof, where it may combine with operators and other rules. The key point is to define the least-level reduction from the very beginning as a reduction firing a redex at minimal level with respect to a general set of redexes (including $\beta_!$, $\beta$ or $\beta_v$, respectively), so that it is "ready" to be extended with other reduction rules (see Section 4).

*Proofs.* All proofs are available in [12], the long version of this paper.

### 2 Background in Abstract Rewriting

An (*abstract*) *rewriting system*, [33, Ch. 1] is a pair $(A, \to)$ consisting of a set $A$ and a binary relation $\to \subseteq A \times A$ (called *reduction*) whose pairs are written $t \to s$ and called *steps*. A $\to$*-sequence* from $t$ is a sequence of $\to$-steps. As usual, $\to^*$ (resp. $\to^=$) denotes the transitive-reflexive (resp. reflexive) closure of $\to$. We say that $u$ is $\to$*-normal* (or a $\to$-normal form) if there is no $t$ such that $u \to t$.

In general, a term may or may not reduce to a normal form. If it does, not all reduction sequences necessarily lead to normal form. A term is *weakly* or *strongly* normalizing, depending on if it may or must reduce to normal form. More precisely, a term $t$ is *strongly* $\to$*-normalizing* if *every* maximal $\to$-sequence from $t$ ends in a $\to$-normal form: any choice of $\to$-steps will eventually lead to a normal form. A term $t$ is *weakly* $\to$*-normalizing* if $t \to^* u$ for some $u$ $\to$-normal. If $t$ is weakly but not strongly normalizing, how do we compute a normal form? This is the problem tackled by *normalization*: by repeatedly performing *only specific steps*, a normal form is eventually reached, provided that $t$ can $\to$-reduce to any.

**Definition 2 (Normalizing and complete strategy).** *A reduction* $\underset{e}{\to} \subseteq \to$ *is a* strategy for $\to$ *if it has the same normal forms as* $\to$*. A strategy* $\underset{e}{\to}$ *for* $\to$ *is:*

- complete *if* $t \underset{e}{\to}^* u$ *whenever* $t \to^* u$ *with* $u$ $\to$*-normal;*
- normalizing *if every weakly* $\to$*-normalizing term is strongly* $\underset{e}{\to}$*-normalizing.*

Note that if the strategy $\underset{e}{\to}$ is complete and *deterministic* (*i.e.* for every $t \in A$, $t \underset{e}{\to} s$ for at most one $s \in A$), then $\underset{e}{\to}$ is a normalizing strategy for $\to$.

Informally, a *strategy* for $\to$ is a way to control the fact that in a term there are different possible choices of a $\to$-step. A *normalizing strategy* for $\to$ is a strategy that is guaranteed to reach a $\to$-normal form, if it exists, from any term. This provides a useful tool to show that a term is not weakly $\to$-normalizing.

**Proving Normalization.** Factorization means that any $\to$-sequence from a term to another can be rearranged by performing a certain kind of steps first. It provides a simple technique to establish that a strategy is normalizing.

**Definition 3 (Factorization).** *Let* $(A, \to)$ *be a rewriting system with* $\to = \underset{e}{\to} \cup \underset{i}{\to}$*. The relation* $\to$ *satisfies* e-factorization*, written* $\mathtt{Fact}(\underset{e}{\to}, \underset{i}{\to})$*, if*

$$\mathtt{Fact}(\underset{e}{\to}, \underset{i}{\to}) : \quad (\underset{e}{\to} \cup \underset{i}{\to})^* \subseteq \underset{e}{\to}^* \cdot \underset{i}{\to}^* \qquad \textbf{(Factorization)}$$

**Lemma 4 (Normalization [2]).** *Let* $\to = \underset{e}{\to} \cup \underset{\neg e}{\to}$*, and* $\underset{e}{\to}$ *be a strategy for* $\to$*.*

*The strategy* $\underset{e}{\to}$ *is* complete *for* $\to$ *if the following conditions hold:*

1. *(*persistence*) if* $t \underset{\neg e}{\to} t'$ *then* $t'$ *is not* $\to$*-normal;*
2. *(*factorization*)* $t \to^* u$ *implies* $t \underset{e}{\to}^* \cdot \underset{\neg e}{\to}^* u$*.*

*The strategy* $\underset{e}{\to}$ *is* normalizing *for* $\to$ *if it is complete and the following holds:*

3. *(*uniformity*) every weakly* $\underset{e}{\to}$*-normalizing term is strongly* $\underset{e}{\to}$*-normalizing.*

A sufficient condition for uniformity (and confluence) is the quasi-diamond.

**Property 5 (Newman [25])** *If a reduction* $\to$ *is* quasi-diamond *(i.e.* $s \leftarrow t \to r$ *implies* $s = r$ *or* $s \to u \leftarrow r$ *for some* $u$*), then* $\to$ *is uniform and confluent (i.e.* $s\,{}^*\!\!\leftarrow r \to^* t$ *implies* $s \to^* u\,{}^*\!\!\leftarrow t$ *for some* $u$*).*

**Proving Factorization.** Hindley [17] first noted that a local property implies factorization. Let $\to = \underset{e}{\to} \cup \underset{i}{\to}$. We say that $\underset{i}{\to}$ *strongly postpones* after $\underset{e}{\to}$ if

$$\mathtt{SP}(\underset{e}{\to}, \underset{i}{\to}) : \qquad \underset{i}{\to} \cdot \underset{e}{\to} \subseteq \underset{e}{\to}^* \cdot \underset{i}{\to}^= \qquad \textbf{(Strong Postponement)}$$

**Lemma 6 (Hindley [17]).** $\mathtt{SP}(\underset{e}{\to}, \underset{i}{\to})$ *implies* $\mathtt{Fact}(\underset{e}{\to}, \underset{i}{\to})$*.*

Strong postponement can rarely be used *directly*, because several interesting reductions—including $\beta$-reduction—do not satisfy it. However, it is at the heart of Takahashi's method [32] to prove head factorization of $\to_\beta$, via the following immediate property that can also be used to prove other factorizations (see [2]).

**Property 7 (Characterization of factorization)** *We have* $\mathtt{Fact}(\underset{\mathsf{e}}{\to}, \underset{\mathsf{i}}{\to})$ *if and only if there is a reduction* $\underset{\mathsf{i}}{\circ\!\!\!\to}$ *such that* $\underset{\mathsf{i}}{\circ\!\!\!\to}^* = \underset{\mathsf{i}}{\to}^*$ *and* $\mathtt{SP}(\underset{\mathsf{e}}{\to}, \underset{\mathsf{i}}{\circ\!\!\!\to})$.

The core of Takahashi's method [32] to prove head factorization in the $\lambda$-calculus is to introduce a relation $\underset{\mathsf{i}}{\Rightarrow}$, called *internal parallel reduction*, which verifies the conditions of Property 7. We will follow a similar path in Section 6.1, to prove *least-level* factorization in the bang calculus.

**Compound systems: proving factorization in a modular way.** In this paper, we will consider compound rewriting systems that are obtained by extending the $\lambda$-calculus with extra rules to model advanced computational features.

In an abstract setting, let us consider a rewrite system $(A, \to)$ where $\to = \to_\xi \cup \to_\rho$. Under which condition $\to$ admits factorization, assuming that both $\to_\xi$ and $\to_\rho$ do? To deal with this question, a technique for proving factorization for *compound systems* in a *modular* way has been introduced in [3]. The approach can be seen as an analogous for factorization of the classical technique for confluence based on Hindley-Rosen lemma [4]: if $\to_\xi, \to_\rho$ are $\mathsf{e}$-factorizing reductions, their union $\to_\xi \cup \to_\rho$ also is, provided that two *local* conditions of commutation hold.

**Lemma 8 (Modular factorization [3]).** *Let* $\to_\xi = \underset{\mathsf{e}}{\to}_\xi \cup \underset{\mathsf{i}}{\to}_\xi$ *and* $\to_\rho = \underset{\mathsf{e}}{\to}_\rho \cup \underset{\mathsf{i}}{\to}_\rho$ *be* $\mathsf{e}$*-factorizing relations. Let* $\underset{\mathsf{e}}{\to} := \underset{\mathsf{e}}{\to}_\xi \cup \underset{\mathsf{e}}{\to}_\rho$ *and* $\underset{\mathsf{i}}{\to} := \underset{\mathsf{i}}{\to}_\xi \cup \underset{\mathsf{i}}{\to}_\rho$*. The reduction* $\to_\xi \cup \to_\rho$ *fulfills factorization* $\mathtt{Fact}(\underset{\mathsf{e}}{\to}, \underset{\mathsf{i}}{\to})$ *if the following swaps hold:*

$$\underset{\mathsf{i}}{\to}_\xi \cdot \underset{\mathsf{e}}{\to}_\rho \subseteq \underset{\mathsf{e}}{\to}_\rho \cdot \to_\xi^* \quad \text{and} \quad \underset{\mathsf{i}}{\to}_\rho \cdot \underset{\mathsf{e}}{\to}_\xi \subseteq \underset{\mathsf{e}}{\to}_\xi \cdot \to_\rho^* \qquad (\textbf{Linear Swaps})$$

The subtlety here is to set $\underset{\mathsf{e}}{\to}_\xi$ and $\underset{\mathsf{e}}{\to}_\rho$ so that $\underset{\mathsf{e}}{\to} = \underset{\mathsf{e}}{\to}_\xi \cup \underset{\mathsf{e}}{\to}_\rho$. As already shown in Example 1, when dealing with normalizing strategies one needs extra care.

### 3 λ-calculi: CbN, CbV, and bang

We present here a generic syntax for $\lambda$-calculi, possibly containing operators. All the variants of the $\lambda$-calculus we shall study use this language. We assume some familiarity with the $\lambda$-calculus, and refer to [4,18] for details.

Given a countable set $\mathsf{Var}$ of variables, denoted by $x, y, z, \ldots$, *terms* and *values* (whose sets are denoted by $\Lambda_{\mathcal{O}}$ and $\mathsf{Val}$, respectively) are defined as follows:

$$t, s, r ::= v \mid ts \mid \mathbf{o}(t_1, \ldots, t_k) \quad \textit{Terms}\colon \Lambda_{\mathcal{O}} \qquad\qquad v ::= x \mid \lambda x.t \quad \textit{Values}\colon \mathsf{Val}$$

where $\mathbf{o}$ ranges over a set $\mathcal{O}$ of function symbols called *operators*, each one with its own arity $k \in \mathbb{N}$. If the operators are $\mathbf{o}_1, \ldots, \mathbf{o}_n$, the set of terms is indicated as $\Lambda_{\mathbf{o}_1\ldots\mathbf{o}_n}$. When the set $\mathcal{O}$ of operators is empty, the calculus is called *pure*, and the sets of terms is denoted by $\Lambda$; otherwise, the calculus is *applied*.

Terms are identified up to renaming of bound variables, where abstraction is the only binder. We denote by $t\{s/x\}$ the capture-avoiding substitution of $s$ for the free occurrences of $x$ in $t$. *Contexts* (with exactly one hole $\langle\cdot\rangle$) are generated by the grammar below, and $\mathbf{c}\langle t\rangle$ stands for the term obtained from the context $\mathbf{c}$ by replacing the hole with the term $t$ (possibly capturing free variables).

$$\mathbf{c} ::= \langle\cdot\rangle \mid t\mathbf{c} \mid \mathbf{c}t \mid \lambda x.\mathbf{c} \mid \mathbf{o}(t_1,\ldots,\mathbf{c},\ldots,t_k) \qquad \textit{Contexts: } \mathcal{C}$$

A *rule* $\rho$ is a binary relation on $\Lambda_{\mathcal{O}}$; we also call it $\rho$-*rule* and denote it by $\mapsto_\rho$, writing $t \mapsto_\rho t'$ rather than $(t,t') \in \rho$. The $\rho$-*reduction* $\to_\rho$ is the contextual closure of $\rho$. Explicitly, $t \to_\rho t'$ holds if $t = \mathbf{c}\langle r\rangle$ and $t' = \mathbf{c}\langle r'\rangle$ for some context $\mathbf{c}$ with $r \mapsto_\rho r'$; the term $r$ is called a $\rho$-*redex*. The set of $\rho$-*redexes* is denoted by $\mathcal{R}_\rho$.

Given a set of rules $\mathsf{Rules}$, the relation $\to\, = \bigcup_\rho \to_\rho$ (for $\rho \in \mathsf{Rules}$) can equivalently be defined as the contextual closure of $\mapsto\, = \bigcup_\rho \mapsto_\rho$.

#### 3.1 Call-by-Name and Call-by-Value $\lambda$-calculi

*Pure CbN and Pure CbV $\lambda$-calculi.* The *pure call-by-name* (CbN for short) $\lambda$-calculus [4,18] is $(\Lambda, \to_\beta)$, the set of terms $\Lambda$ together with the $\beta$-reduction $\to_\beta$, defined as the contextual closure of the usual $\beta$-rule, which we recall in (1) below.

The *pure call-by-value* (CbV for short) $\lambda$-calculus [27] is the set $\Lambda$ endowed with the reduction $\to_{\beta_v}$, defined as the contextual closure of the $\beta_v$-rule in (2).

$$\text{CbN: } (\lambda x.t)s \mapsto_\beta t\{s/x\} \quad (1) \qquad \text{CbV: } (\lambda x.t)v \mapsto_{\beta_v} t\{v/x\} \;\; \text{with } v \in \mathsf{Val} \quad (2)$$

*CbN and CbV $\lambda$-calculi.* A *CbN* (resp. *CbV*) $\lambda$-*calculus* is the set of terms endowed with a reduction $\to$ which extends $\to_\beta$ (resp. $\to_{\beta_v}$).

In particular, the *applied* setting with operators (when $\mathcal{O} \neq \emptyset$) models in the $\lambda$-calculus richer computational features, allowing $\mathbf{o}$-reductions as the contextual closure of $\mathbf{o}$-rules of the form $\mathbf{o}(t_1,\ldots,t_k) \mapsto_{\mathbf{o}} s$.

*Example 9 (Non-deterministic $\lambda$-calculi).* Let $\mathcal{O} = \{\oplus\}$ where $\oplus$ is a binary operator; let $\to_\oplus$ be the contextual closure of the (non-deterministic) rule below:

$$\oplus(t_1,t_2) \mapsto_\oplus t_1 \quad \text{and} \quad \oplus(t_1,t_2) \mapsto_\oplus t_2.$$

The *non-deterministic CbN $\lambda$-calculus* $\Lambda_\oplus^{\mathtt{cbn}} = (\Lambda_\oplus, \to_{\beta\oplus})$ is the set $\Lambda_\oplus$ with the reduction $\to_{\beta\oplus} = \to_\beta \cup \to_\oplus$. The *non-deterministic CbV $\lambda$-calculus* $\Lambda_\oplus^{\mathtt{cbv}} = (\Lambda_\oplus, \to_{\beta_v\oplus})$ is the set $\Lambda_\oplus$ with the reduction $\to_{\beta_v\oplus} = \to_{\beta_v} \cup \to_\oplus$.

#### 3.2 Bang calculi

The bang calculus [11,15] is a variant of the $\lambda$-calculus inspired by linear logic. An operator ! plays the role of a marker for duplicability and discardability. Here we allow also the presence of operators other than !, ranging over a set $\mathcal{O}$. So, terms and contexts of the bang calculus (denoted by capital letters) are:

$$T, S, R ::= x \mid \lambda x.T \mid TS \mid !T \mid \mathbf{o}(T_1, \dots, T_k) \qquad \textit{Terms: } \Lambda_{!\mathcal{O}}$$
$$\mathbf{C} ::= \langle\cdot\rangle \mid \lambda x.\mathbf{C} \mid T\mathbf{C} \mid \mathbf{C}T \mid !\mathbf{C} \mid \mathbf{o}(T_1, \dots, \mathbf{C}, \dots, T_k) \qquad \textit{Contexts: } \mathcal{C}_!$$

Terms of the form $!T$ are called *boxes* and their set is denoted by $!\Lambda_{!\mathcal{O}}$. When there are no operators other than ! (*i.e.* $\mathcal{O} = \emptyset$), the set of terms and the set of boxes are denoted by $\Lambda_!$ and $!\Lambda_!$, respectively. This syntax can be expressed in the one at the beginning of Section 3, where ! is an unary operator called *bang*.

*The pure bang calculus.* The *pure* bang calculus $(\Lambda_!, \to_{\beta_!})$ is the set of terms $\Lambda_!$ endowed with reduction $\to_{\beta_!}$, the closure under contexts in $\mathcal{C}_!$ of the $\beta_!$-*rule*:

$$(\lambda x.T)\,!S \mapsto_{\beta_!} T\{S/x\} \tag{3}$$

Intuitively, in the bang calculus the bang-operator ! marks the only terms that can be erased and duplicated. Indeed, a *β-like redex* $(\lambda x.T)S$ can be fired by $\mapsto_{\beta_!}$ only when its argument $S$ is a box, *i.e.* $S = !R$: if it is so, the content $R$ of the box $S$ (and not $S$ itself) replaces any free occurrence of $x$ in $T$.[3]

A proof of confluence of $\beta_!$-reduction $\to_{\beta_!}$ is in [15].

**Notation 10** *We use the following notations to denote some notable terms.*

$$\iota := \lambda x.x \qquad \delta := \lambda x.xx \qquad I := \lambda x.!x \qquad \Delta := \lambda x.x\,!x.$$

*Remark 11 (Notable terms).* The term $I = \lambda x.!x$ plays the role of the identity in the bang calculus: $I\,!T \to_{\beta_!} !(x\{T/x\}) = !T$ for any term $T$. Instead, the term $\iota = \lambda x.x$, when applied to a box $!T$, opens the box, *i.e.* returns its content $T$: $\iota\,!T \to_{\beta_!} x\{T/x\} = T$. Finally, $\Delta\,!\Delta \to_{\beta_!} \Delta\,!\Delta \to_{\beta_!} \dots$ is a diverging term.

*A bang calculus.* A *bang calculus* $(\Lambda_{!\mathcal{O}}, \to)$ is the set $\Lambda_{!\mathcal{O}}$ of terms endowed with a reduction $\to$ which extends $\to_{\beta_!}$. In this paper we shall consider calculi where $\to$ contains $\to_{\beta_!}$ and $\mathbf{o}$-reductions $\to_{\mathbf{o}}$ ($\mathbf{o} \in \mathcal{O}$) defined from $\mathbf{o}$-rules of the form $\mathbf{o}(T_1, \dots, T_k) \mapsto_{\mathbf{o}} S$, and possibly other rules. So, $\to = \bigcup_\rho \to_\rho$ (for $\rho \in \mathsf{Rules}$), with $\mathsf{Rules} \supseteq \{!\beta, \mathbf{o} \mid \mathbf{o} \in \mathcal{O}\}$. We set $\to_{\mathcal{O}} = \bigcup_{\mathbf{o}\in\mathcal{O}} \to_{\mathbf{o}}$.

#### 3.3 CbN and CbV translations into the bang calculus

Our motivation to study the bang calculus is to have a general framework where both CbN [4] and CbV [27] λ-calculi can be embedded, via two distinct translations. Here we show how these translations work. We extend the simulation results in [15,30,7] for the pure case to the case with operators (Proposition 13).

Following [7], the CbV translation defined here differs from [15,30] in the application case. Section 5 will show why this optimization is crucial.

*CbN* and *CbV translations* are two maps $(\cdot)^{\mathsf{n}} \colon \Lambda_{\mathcal{O}} \to \Lambda_{!\mathcal{O}}$ and $(\cdot)^{\mathsf{v}} \colon \Lambda_{\mathcal{O}} \to \Lambda_{!\mathcal{O}}$, respectively, translating terms of the λ-calculus into terms of the bang calculus:

[3] Syntax and reduction rule of the bang calculus follow [15], which is slightly different from [11]. Unlike [15] (but akin to [30,16]), here we do not use $\iota$ (aka $\mathsf{der}$) as a primitive, since $\iota$ and its associated rule $\mapsto_{\mathsf{d}}$ can be simulated, see Remark 11 and (4).

$$x^{\mathsf{n}} = x \quad (\lambda x.t)^{\mathsf{n}} = \lambda x.t^{\mathsf{n}} \quad (\mathbf{o}(t_1,\dots,t_k))^{\mathsf{n}} = \mathbf{o}(t_1^{\mathsf{n}},\dots,t_k^{\mathsf{n}}) \quad (ts)^{\mathsf{n}} = t^{\mathsf{n}}\,!s^{\mathsf{n}}\,;$$
$$x^{\mathsf{v}} = !x \quad (\lambda x.t)^{\mathsf{v}} = !(\lambda x.t^{\mathsf{v}}) \quad (\mathbf{o}(t_1,\dots,t_k))^{\mathsf{v}} = \mathbf{o}(t_1^{\mathsf{v}},\dots,t_k^{\mathsf{v}}) \quad (ts)^{\mathsf{v}} = \begin{cases} T\,s^{\mathsf{v}} & \text{if } t^{\mathsf{v}} = !T \\ (\iota\,t^{\mathsf{v}})s^{\mathsf{v}} & \text{otherwise.} \end{cases}$$

*Example 12.* Consider the $\lambda$-term $\omega \coloneqq \delta\delta$: then, $\delta^{\mathsf{n}} = \Delta$, $\delta^{\mathsf{v}} = !\Delta$ and $\omega^{\mathsf{n}} = \Delta\,!\Delta = \omega^{\mathsf{v}}$ ($\delta$ and $\Delta$ are defined in Notation 10). The $\lambda$-term $\omega$ is diverging in CbN and CbV $\lambda$-calculi, and so is $\omega^{\mathsf{n}} = \omega^{\mathsf{v}}$ in the bang calculus, see Remark 11.

For any term $t \in \Lambda_{\mathcal{O}}$, $t^{\mathsf{n}}$ and $t^{\mathsf{v}}$ are just different decorations of $t$ by means of the bang-operator ! (recall that $\iota = \lambda x.x$). The translation $(\cdot)^{\mathsf{n}}$ puts the argument of any application into a box: in CbN any term is duplicable or discardable. On the other hand, only *values* (*i.e.* abstractions and variables) are translated by $(\cdot)^{\mathsf{v}}$ into boxes, as they are the only terms duplicable or discardable in CbV.

As in [15,30], we prove that the CbN translation $(\cdot)^{\mathsf{n}}$ (resp. CbV translation $(\cdot)^{\mathsf{v}}$) from the pure CbN (resp. CbV) $\lambda$-calculus into the bang calculus is *sound* and *complete*: it maps $\beta$-reductions (resp. $\beta_v$-reductions) of the $\lambda$-calculus into $\beta_!$-reductions of the bang calculus, and conversely $\beta_!$-reductions — when restricted to the image of the translation — into $\beta$-reductions (resp. $\beta_v$-reductions). The same holds if we consider any $\mathbf{o}$-reduction for operators, where we assume that the $\mathbf{o}$-rule commutes with the translations: if $\mathbf{o}(t_1,\dots,t_k) \mapsto_{\mathbf{o}} s$ then $\mathbf{o}(t_1^{\mathsf{n}},\dots,t_k^{\mathsf{n}}) \mapsto_{\mathbf{o}} s^{\mathsf{n}}$, and if $\mathbf{o}(t_1^{\mathsf{n}},\dots,t_k^{\mathsf{n}}) \mapsto_{\mathbf{o}} S$ then $\mathbf{o}(t_1,\dots,t_k) \mapsto_{\mathbf{o}} s$ with $s^{\mathsf{n}} = S$; similarly for $(\cdot)^{\mathsf{v}}$.

In the simulation, $\rightarrow_{\mathsf{d}}$ denotes the contextual closure of the rule:

$$\iota\,!T \mapsto_{\mathsf{d}} T \quad \text{(this is nothing but } (\lambda x.x)!T \mapsto_{\beta_!} T\text{)} \tag{4}$$

Clearly, $\rightarrow_{\mathsf{d}} \subseteq \rightarrow_{\beta_!}$ (Remark 11). We write $T \twoheadrightarrow_{\mathsf{d}} S$ if $T \rightarrow_{\mathsf{d}}^* S$ and $S$ is $\mathsf{d}$-normal.

**Proposition 13 (Simulation of CbN and CbV).** *Let $t \in \Lambda_{\mathcal{O}}$ and $\mathbf{o} \in \mathcal{O}$.*

1. CbN soundness: *If $t \rightarrow_{\beta} t'$ then $t^{\mathsf{n}} \rightarrow_{\beta_!} t'^{\mathsf{n}}$. If $t \rightarrow_{\mathbf{o}} t'$ then $t^{\mathsf{n}} \rightarrow_{\mathbf{o}} t'^{\mathsf{n}}$.*
   CbN completeness: *If $t^{\mathsf{n}} \rightarrow_{\beta_!} S$ then $S = t'^{\mathsf{n}}$ and $t \rightarrow_{\beta} t'$, for some $t' \in \Lambda_{\mathcal{O}}$. If $t^{\mathsf{n}} \rightarrow_{\mathbf{o}} S$ then $S = t'^{\mathsf{n}}$ and $t \rightarrow_{\mathbf{o}} t'$, for some $t' \in \Lambda_{\mathcal{O}}$.*
2. CbV soundness: *If $t \rightarrow_{\beta_v} t'$ then $t^{\mathsf{v}} \rightarrow_{\beta_!}\rightarrow_{\mathsf{d}}^{=} t'^{\mathsf{v}}$ with $t'^{\mathsf{v}}$ $\mathsf{d}$-normal. If $t \rightarrow_{\mathbf{o}} t'$ then $t^{\mathsf{v}} \rightarrow_{\mathbf{o}}\rightarrow_{\mathsf{d}}^{=} t'^{\mathsf{v}}$ with $t'^{\mathsf{v}}$ $\mathsf{d}$-normal.*
   CbV completeness: *If $t^{\mathsf{v}} \rightarrow_{\beta_!}\twoheadrightarrow_{\mathsf{d}} S$ then $t^{\mathsf{v}} \rightarrow_{\beta_!}\rightarrow_{\mathsf{d}}^{=} S$ with $S = t'^{\mathsf{v}}$ and $t \rightarrow_{\beta_v} t'$, for some $t' \in \Lambda_{\mathcal{O}}$. If $t^{\mathsf{v}} \rightarrow_{\mathbf{o}}\twoheadrightarrow_{\mathsf{d}} S$ then $t^{\mathsf{v}} \rightarrow_{\mathbf{o}}\rightarrow_{\mathsf{d}}^{=} S$ with $S = t'^{\mathsf{v}}$ and $t \rightarrow_{\mathbf{o}} t'$, for some $t' \in \Lambda_{\mathcal{O}}$.*

*Example 14.* Let $t = (\lambda z.z)x\,y$ and $t' = xy$. So $t \rightarrow_{\beta} t'$ with $t^{\mathsf{n}} = (\lambda z.z)!x\,!y \rightarrow_{\beta_!} x\,!y = t'^{\mathsf{n}}$; and $t \rightarrow_{\beta_v} t'$ with $t^{\mathsf{v}} = (\iota((\lambda z.!z)!x))!y \rightarrow_{\beta_!} (\iota\,!x)!y \rightarrow_{\mathsf{d}} x\,!y = t'^{\mathsf{v}}$.

### 4 The least-level strategy

The bang calculus $\Lambda_!$ has a natural normalizing strategy, derived from linear logic [8], namely the *least-level reduction*. It reduces only redexes at *least level*, where the *level* of a redex $R$ in a term $T$ is the number of bangs ! in which $R$ is nested.

Least-level reduction is easily extended to a general bang calculus $(\Lambda_{!\mathcal{O}}, \to)$. The level of a redex $R$ is then the number of bangs ! and operators $\mathbf{o}$ in which $R$ is nested; intuitively, least-level reduction fires a redex which is *minimally nested*.

Below, we formalize the reduction in a way that is independent of the specific shape of the redexes, and even of specific definition of level one chooses. The interest of least-level reduction is in the properties it satisfies. All our developments will rely on such properties, rather than the specific definition of least level.

In this section, $\to = \bigcup_\rho \to_\rho$ for $\rho \in \mathsf{Rules}$ (for a generic set of rules $\mathsf{Rules}$). We write $\mathcal{R} = \bigcup_\rho \mathcal{R}_\rho$ (again, with $\rho \in \mathsf{Rules}$) for the set of *all* redexes.

#### 4.1 Least-level reduction in bang calculi

The *level* of a redex occurrence $R$ in a term $T$ is a measure of its depth. Formally, we indicate the *occurrence of a subterm* $R$ in $T$ with the context $\mathbf{C}$ such that $\mathbf{C}\langle R\rangle = T$. Its level is then the *level* $\ell(\mathbf{C}) \in \mathbb{N}$ of the hole in $\mathbf{C}$. The definition of *level* for contexts in a bang calculus $\Lambda_{!\mathcal{O}}$ is formalized as follows.

$$\begin{gathered}\ell(\langle\cdot\rangle) = 0 \qquad \ell(\lambda x.\mathbf{C}) = \ell(\mathbf{C}) \qquad \ell(\mathbf{C}T) = \ell(\mathbf{C}) \qquad \ell(T\mathbf{C}) = \ell(\mathbf{C}) \\ \ell(!\mathbf{C}) = \ell(\mathbf{C}) + 1 \qquad \ell(\mathbf{o}(\dots, \mathbf{C}, \dots)) = \ell(\mathbf{C}) + 1\end{gathered} \tag{5}$$

Note that the level increases by 1 in the scope of !, and of any operator $\mathbf{o} \in \mathcal{O}$.

A reduction step $T \to_\rho S$ is *at level* $k$ if it fires a $\rho$-redex at level $k \in \mathbb{N}$; it is *least-level* if it reduces a redex whose level is minimal.

The *least level* $\ell\ell(T)$ of a term $T$ expresses the minimal level of any redex occurrences in $T$; if no redex is in $T$, we set $\ell\ell(T) = \infty$. Formally:

**Definition 15 (Least-level reduction).** *Let* $\to = \bigcup_\rho \to_\rho$ *(for* $\rho \in \mathsf{Rules}$*) and* $\mathcal{R} = \bigcup_\rho \mathcal{R}_\rho$ *the set of redexes. Given a function* $\ell(\cdot)$ *from contexts to* $\mathbb{N}$*:*

- *The* least level *of a term* $T$ *is defined as*[4]

$$\ell\ell(T) \coloneqq \inf\{\ell(\mathbf{C}) \mid T = \mathbf{C}\langle R\rangle \text{ for some } R \in \mathcal{R}\} \in (\mathbb{N} \cup \{\infty\}). \tag{6}$$

- *A* $\rho$*-reduction step* $T \to_\rho S$ *is:*
  1. at level $k$*, noted* $T \to_{\rho:k} S$*, if* $T = \mathbf{C}\langle R\rangle$*,* $S = \mathbf{C}\langle R'\rangle$*,* $R \mapsto_\rho R'$*,* $\ell(\mathbf{C}) = k$*;*
  2. least-level*, noted* $T \underset{\mathrm{L}}{\to}_\rho S$*, if* $T \to_{\rho:k} S$ *and* $k = \ell\ell(T)$*;*
  3. internal*, noted* $T \underset{\neg\mathrm{L}}{\to}_\rho S$*, if* $T \to_{\rho:k} S$ *and* $k > \ell\ell(T)$*.*
- Least-level reduction *is* $\underset{\mathrm{L}}{\to} = \bigcup_\rho \underset{\mathrm{L}}{\to}_\rho$ *(for* $\rho \in \mathsf{Rules}$*).*
- Internal reduction *is* $\underset{\neg\mathrm{L}}{\to} = \bigcup_\rho \underset{\neg\mathrm{L}}{\to}_\rho$ *(for* $\rho \in \mathsf{Rules}$*).*

Note that $\to\, = \underset{\mathrm{L}}{\to} \cup \underset{\neg\mathrm{L}}{\to}$ and that our definitions solve the issue of Example 1. Indeed, the definition of least level $\ell\ell(T)$ of a term, and hence the definition of $\underset{\mathrm{L}}{\to}_\rho$, depend on the *whole* set $\mathcal{R} = \bigcup_\rho \mathcal{R}_\rho$ of redexes associated with $\to$.[5]

[4] Recall that $\inf \emptyset = \infty$, when $\emptyset$ is seen as the empty subset of $\mathbb{N}$ with the usual order.
[5] We should write $\ell\ell_{\mathcal{R}}(T)$, $\mathrm{L}_{\mathcal{R}}$ and $\underset{\mathrm{L}_{\mathcal{R}}}{\to}_\rho$, but we avoid it for the sake of readability.

*Normal Forms.* It is immediate that $\underset{L}{\rightarrow} \subsetneq \rightarrow$ is a *strategy* for $\rightarrow$. Indeed, $\underset{L}{\rightarrow}$ and $\rightarrow$ have the *same normal forms* because $\underset{L}{\rightarrow} \subseteq \rightarrow$ and if a term has a $\rightarrow$-redex, it has a redex at least-level, *i.e.* it has a $\underset{L}{\rightarrow}$-redex.

*Remark 16 (Least level of normal forms).* Note that $\ell\ell(T) = \infty$ if and only if $T$ is $\rightarrow$-normal, because $\ell(\mathbf{C}) \in \mathbb{N}$ for all contexts $\mathbf{C}$.

*A good least-level reduction.* The beauty of least-level reduction for the bang calculus, is that it satisfies some elegant properties, which allow for neat proofs, in particular monotonicity and internal invariance (in Definition 17). The developments in the rest of the paper rely on such properties, and in fact will apply to any calculus whose reduction $\rightarrow$ has the properties described below.

**Definition 17 (Good least-level).** *A reduction $\rightarrow$ has a* good least-level *if:*

1. *(*monotonicity*) $T \rightarrow S$ implies $\ell\ell(T) \leq \ell\ell(S)$; and*
2. *(*internal invariance*) $T \underset{\neg L}{\rightarrow} S$ implies $\ell\ell(T) = \ell\ell(S)$.*

Point 1 states that no step can decrease the least level of a term. Point 2 says that internal steps cannot change the least level of a term. Therefore, only least-level steps may increase the least level. Together, they imply persistence: only least-level steps can approach normal forms.

**Property 18 (Persistence)** *If $\rightarrow$ has a good least-level, then $T \underset{\neg L}{\rightarrow} S$ implies that $S$ is not $\rightarrow$-normal.*

Reduction $\rightarrow_{\beta_!}$ in the pure bang calculus $(\Lambda_!, \rightarrow_{\beta_!})$ has a good least-level. More in general, the same holds when extending the reduction with operators.

**Proposition 19 (Good least-level of bang calculi).** *Given $\Lambda_{!\mathcal{O}}$, let $\rightarrow = \rightarrow_{\beta_!} \cup \rightarrow_{\mathcal{O}}$, where each $\mathbf{o} \in \mathcal{O}$ has a redex of shape $\mathbf{o}(P_1, \ldots, P_k)$. The reduction $\rightarrow$ has a good least-level.*

#### 4.2 Least-level for a bang calculus: examples.

Let us see more closely the least-level reduction for a bang calculus $(\Lambda_{!\mathcal{O}}, \rightarrow)$. For concreteness, we consider $\mathsf{Rules} = \{\beta_!, \mathbf{o} \mid \mathbf{o} \in \mathcal{O}\}$, hence the set of redexes is $\mathcal{R} = \mathcal{R}_{\beta_!} \cup \mathcal{R}_{\mathcal{O}}$, where $\mathcal{R}_{\mathcal{O}}$ is the set of terms $\mathbf{o}(T_1, \ldots, T_k)$ for any $\mathbf{o} \in \mathcal{O}$.

We observe that the least level $\ell\ell(T)$ of a term $T \in \Lambda_{!\mathcal{O}}$ can be easily defined in a direct way, by induction on $T$:

- $\ell\ell(T) = 0$ if $T \in \mathcal{R} = \mathcal{R}_{\beta_!} \cup \mathcal{R}_{\mathcal{O}}$,
- otherwise, $\ell\ell(x) = \infty$ and

$$\ell\ell(\lambda x.T) = \ell\ell(T) \qquad \ell\ell(!T) = \ell\ell(T) + 1 \qquad \ell\ell(TS) = \min\{\ell\ell(T), \ell\ell(S)\}.$$

*Example 20 (Least level of a term).* Let $R \in \mathcal{R}_{\beta_!}$. If $T_0 := R\,!R$, then $\ell\ell(T_0) = 0$. If $T_1 := x\,!R$ then $\ell\ell(T_1) = 1$. If $T_2 := \mathbf{o}(x, y)!R$ then $\ell\ell(T_2) = 0$, as $\mathbf{o}(x, y) \in \mathcal{R}_{\mathcal{O}}$.

Intuitively, least-level reduction fires a redex that is *minimally nested*, where a redex is any subterm whose form is in $\mathcal{R} = \mathcal{R}_{\beta_!} \cup \mathcal{R}_{\mathcal{O}}$. Note that least-level reduction can choose to fire one among possibly *several* redexes at minimal level.

*Example 21.* Let us revisit Example 20 with $R = \iota\,!z \in \mathcal{R}_{\beta_!}$ (so $R \mapsto_{\beta_!} z$, see Remark 11). Then $T_1 \coloneqq x\,!R \underset{\mathrm{L}}{\to}_{\beta_!} x\,!z$ but $T_0 \coloneqq R\,!R \underset{\mathrm{L}}{\not\to}_{\beta_!} R!z$ and $T_2 \coloneqq \mathbf{o}(x,y)\,!R \underset{\mathrm{L}}{\not\to}_{\beta_!} \mathbf{o}(x,y)!z$. Also, $\mathbf{o}(x,R) \underset{\mathrm{L}}{\not\to}_{\beta_!} \mathbf{o}(x,z)$ although $\mathbf{o}(x,R) \to_{\beta_!} \mathbf{o}(x,z)$.

Let $S = \iota\,!(z\,!z)$ (so $S \mapsto_{\beta_!} z\,!z$). In $(\lambda z.S)!S$, two least-level steps are possible (the fired $\beta_!$-redex is underlined): $\underline{(\lambda z.S)!S} \underset{\mathrm{L}}{\to}_{\beta_!} \iota\,!(S\,!S)$, and $(\lambda z.\underline{S})!S \underset{\mathrm{L}}{\to}_{\beta_!} (\lambda z.z\,!z)!S$. But $(\lambda z.S)!S \underset{\mathrm{L}}{\not\to}_{\beta_!} (\lambda z.S)!(z\,!z)$ although $(\lambda z.S)!S \to_{\beta_!} (\lambda z.S)!(z\,!z)$.

#### 4.3 Least-level for CbN and CbV λ-calculi

The definition of least-level reduction in Section 4.1 is independent of the specific notion of level chosen, and of the specific calculus. The idea is that the reduction strategy persistently fires a redex at minimal level, once such a notion is set.

Least-level reduction can indeed be defined also for the CbN and CbV λ-calculi, given an opportune definition of level. In CbN, we count the number of nested arguments and operators containing the redex occurrence. In CbV, we count the number of nested operators and *unapplied* abstractions containing the redex occurrence, where an abstraction is unapplied if it is not the right-hand side of an application. Formally, a redex occurrence is identified by a context (as explained in Section 4.1), and we define the *level* $\ell^{\mathrm{CbN}}(\mathbf{c}) \in \mathbb{N}$ and $\ell^{\mathrm{CbV}}(\mathbf{c}) \in \mathbb{N}$ of a context $\mathbf{c}$ in CbN and CbV λ-calculi, respectively, as follows.

$$
\begin{aligned}
\ell^{\mathrm{CbN}}(\langle\cdot\rangle) &= 0 & \ell^{\mathrm{CbV}}(\langle\cdot\rangle) &= 0\\
\ell^{\mathrm{CbN}}(\lambda x.\mathbf{c}) &= \ell^{\mathrm{CbN}}(\mathbf{c}) & \ell^{\mathrm{CbV}}(\lambda x.\mathbf{c}) &= \ell^{\mathrm{CbV}}(\mathbf{c}) + 1\\
\ell^{\mathrm{CbN}}(\mathbf{c}t) &= \ell^{\mathrm{CbN}}(\mathbf{c}) & \ell^{\mathrm{CbV}}(\mathbf{c}t) &= \begin{cases}\ell^{\mathrm{CbV}}(\mathbf{c}') & \text{if } \mathbf{c} = \lambda x.\mathbf{c}'\\ \ell^{\mathrm{CbV}}(\mathbf{c}) & \text{otherwise}\end{cases}\\
\ell^{\mathrm{CbN}}(t\mathbf{c}) &= \ell^{\mathrm{CbN}}(\mathbf{c}) + 1 & \ell^{\mathrm{CbV}}(t\mathbf{c}) &= \ell^{\mathrm{CbV}}(\mathbf{c})\\
\ell^{\mathrm{CbN}}(\mathbf{o}(\dots,\mathbf{c},\dots)) &= \ell^{\mathrm{CbN}}(\mathbf{c}) + 1 & \ell^{\mathrm{CbV}}(\mathbf{o}(\dots,\mathbf{c},\dots)) &= \ell^{\mathrm{CbV}}(\mathbf{c}) + 1.
\end{aligned}
$$

In both CbN and CbV λ-calculi, the *least level* of a term (denoted by $\ell\ell^{\mathrm{CbN}}(\cdot)$ and $\ell\ell^{\mathrm{CbV}}(\cdot)$) and *least-level* and *internal* reductions are given by Definition 15 (replace $\ell(\cdot)$ with $\ell^{\mathrm{CbN}}(\cdot)$ for CbN, and with $\ell^{\mathrm{CbV}}(\cdot)$ for CbV).

In Section 5 we will see that the definitions of CbN and CbV least level are not arbitrary, but induced by the CbN and CbV translations defined in Section 3.3.

### 5 Embedding of CbN and CbV by level

Here we refine the analysis of the CbN and CbV translations given in Section 3.3, by showing two new results: translations preserve normal forms (Proposition 22) and least-level (Proposition 25), back and forth. This way, to obtain least-level

*factorization* or least-level *normalization* results, it suffices to prove them in the bang calculus. The translation transfers the results into the CbN and CbV $\lambda$-calculi (Theorem 26). We use here the expression "translate" in a strong sense: the results for CbN and CbV $\lambda$-calculi are obtained from the corresponding results in the bang calculus almost for free, just via CbN and CbV translations.

*Preservation of normal forms.* The targets of the CbN translation $(\cdot)^{\mathsf{n}}$ and CbV translation $(\cdot)^{\mathsf{v}}$ into the bang calculus can be *characterized syntactically.* A fine analysis of these fragments of the bang calculus (see [12] for details) proves that both CbN and CbV translations preserve normal forms, back and forth.

**Proposition 22 (Preservation of normal forms).** *Let $t, s \in \Lambda_{\mathcal{O}}$ and $\mathbf{o} \in \mathcal{O}$.*

1. CbN: *$t$ is $\beta$-normal iff $t^{\mathsf{n}}$ is $\beta_!$-normal; $t$ is $\mathbf{o}$-normal iff $t^{\mathsf{n}}$ is $\mathbf{o}$-normal.*
2. CbV: *$t$ is $\beta_v$-normal iff $t^{\mathsf{v}}$ is $\beta_!$-normal; $t$ is $\mathbf{o}$-normal iff $t^{\mathsf{v}}$ is $\mathbf{o}$-normal.*

By Remark 16, Proposition 22 can be seen as the fact that CbN and CbV translations preserve the least-level of a term, back and forth, when the least-level is infinite. Actually, this holds more in general for any value of the least-level.

*Preservation of levels.* We aim to show that least-level steps in CbN and CbV $\lambda$-calculi correspond to least-level steps in the bang calculus—back and forth—via CbN and CbV translations, respectively (Proposition 25). This result is subtle, one of the main technical contributions of this paper.

First, we extend the definition of translations to contexts. The *CbN and CbV translations for contexts* are two functions $(\cdot)^{\mathsf{n}} : \mathcal{C} \to \mathcal{C}_!$ and $(\cdot)^{\mathsf{v}} : \mathcal{C} \to \mathcal{C}_!$, respectively, mapping contexts of the $\lambda$-calculus into contexts of the bang calculus:

$$\begin{aligned} \langle\cdot\rangle^{\mathsf{n}} &= \langle\cdot\rangle \\ (\lambda x.\mathbf{c})^{\mathsf{n}} &= \lambda x.\mathbf{c}^{\mathsf{n}} \\ (\mathbf{o}(t_1,\dots,\mathbf{c},\dots,t_k))^{\mathsf{n}} &= \mathbf{o}(t_1^{\mathsf{n}},\dots,\mathbf{c}^{\mathsf{n}},\dots,t_k^{\mathsf{n}}) \\ (\mathbf{c}t)^{\mathsf{n}} &= \mathbf{c}^{\mathsf{n}}\,!(t^{\mathsf{n}}) \\ (t\mathbf{c})^{\mathsf{n}} &= t^{\mathsf{n}}\,!(\mathbf{c}^{\mathsf{n}})\,; \end{aligned} \qquad \begin{aligned} \langle\cdot\rangle^{\mathsf{v}} &= \langle\cdot\rangle \\ (\lambda x.\mathbf{c})^{\mathsf{v}} &= !(\lambda x.\mathbf{c}^{\mathsf{v}}) \\ (\mathbf{o}(t_1,\dots,\mathbf{c},\dots,t_k))^{\mathsf{v}} &= \mathbf{o}(t_1^{\mathsf{v}},\dots,\mathbf{c}^{\mathsf{v}},\dots,t_k^{\mathsf{v}}) \\ (\mathbf{c}t)^{\mathsf{v}} &= \begin{cases} \mathbf{C}\,t^{\mathsf{v}} & \text{if } \mathbf{c}^{\mathsf{v}} = !\mathbf{C} \\ (\iota\,\mathbf{c}^{\mathsf{v}})t^{\mathsf{v}} & \text{otherwise} \end{cases} \\ (t\mathbf{c})^{\mathsf{v}} &= \begin{cases} T\,\mathbf{c}^{\mathsf{v}} & \text{if } t^{\mathsf{v}} = !T \\ (\iota\,t^{\mathsf{v}})\mathbf{c}^{\mathsf{v}} & \text{otherwise.} \end{cases} \end{aligned}$$

Note that CbN (resp. CbV) level of a context defined in Section 4.3 increases by 1 whenever the CbN (resp. CbV) translation for contexts adds a !. Thus, CbN and CbV translations preserve, back and forth, the level of a redex and the least-level of a term. Said differently, the level for CbN and CbV is defined in Section 4.3 so as to enable the preservation of level via CbN and CbV translations.

**Lemma 23 (Preservation of level via CbN translation).**

1. For contexts: *For any context $\mathbf{c} \in \mathcal{C}$, one has $\ell^{\mathrm{CbN}}(\mathbf{c}) = \ell(\mathbf{c}^{\mathsf{n}})$.*
2. For reduction: *For any term $t \in \Lambda_{\mathcal{O}}$: $t \to_{\beta:k} s$ if and only if $t^{\mathsf{n}} \to_{\beta_!:k} s^{\mathsf{n}}$; and $t \to_{\mathbf{o}:k} s$ if and only if $t^{\mathsf{n}} \to_{\mathbf{o}:k} s^{\mathsf{n}}$, for any $\mathbf{o} \in \mathcal{O}$.*

3. For least-level of a term: *For any term* $t \in \Lambda_{\mathcal{O}}$*, one has* $\ell\ell^{\mathrm{CbN}}(t) = \ell\ell(t^{\mathsf{n}})$*.*

**Lemma 24 (Preservation of level via CbV translation).**

1. For contexts: *For any context* $\mathbf{c} \in \mathcal{C}$*, one has* $\ell^{\mathrm{CbV}}(\mathbf{c}) = \ell(\mathbf{c}^{\mathsf{v}})$*.*
2. For reduction: *For any term* $t \in \Lambda_{\mathcal{O}}$*:* $t \to_{\beta_v : k} s$ *if and only if* $t^{\mathsf{v}} \to_{\beta_! : k} \to_{\mathsf{d}:k}^{=} s^{\mathsf{v}}$*; and* $t \to_{\mathbf{o}:k} s$ *if and only if* $t^{\mathsf{v}} \to_{\mathbf{o}:k} \to_{\mathsf{d}:k}^{=} s^{\mathsf{v}}$*, for any* $\mathbf{o} \in \mathcal{O}$*.*
3. For least-level of a term: *For any term* $t \in \Lambda_{\mathcal{O}}$*, one has* $\ell\ell^{\mathrm{CbV}}(t) = \ell\ell(t^{\mathsf{v}})$*.*

From the two lemmas above it follows that CbN and CbV translations preserve least-level and internal reductions, back and forth.

**Proposition 25 (Preservation of least-level and internal reductions).** *Let* $t \in \Lambda_{\mathcal{O}}$ *and* $\mathbf{o} \in \mathcal{O}$*.*

1. CbN least-level: $t \underset{\mathrm{L}}{\to}_{\beta} s$ *iff* $t^{\mathsf{n}} \underset{\mathrm{L}}{\to}_{\beta_!} s^{\mathsf{n}}$*; and* $t \underset{\mathrm{L}}{\to}_{\mathbf{o}} s$ *iff* $t^{\mathsf{n}} \underset{\mathrm{L}}{\to}_{\mathbf{o}} s^{\mathsf{n}}$*.*
2. CbN internal: $t \underset{\neg\mathrm{L}}{\to}_{\beta} s$ *iff* $t^{\mathsf{n}} \underset{\neg\mathrm{L}}{\to}_{\beta_!} s^{\mathsf{n}}$*; and* $t \underset{\neg\mathrm{L}}{\to}_{\mathbf{o}} s$ *iff* $t^{\mathsf{n}} \underset{\neg\mathrm{L}}{\to}_{\mathbf{o}} s^{\mathsf{n}}$*.*
3. CbV least-level: $t \underset{\mathrm{L}}{\to}_{\beta_v} s$ *iff* $t^{\mathsf{v}} \underset{\mathrm{L}}{\to}_{\beta_!} \underset{\mathrm{L}}{\to}_{\mathsf{d}}^{=} s^{\mathsf{v}}$*; and* $t \underset{\mathrm{L}}{\to}_{\mathbf{o}} s$ *iff* $t^{\mathsf{v}} \underset{\mathrm{L}}{\to}_{\mathbf{o}} \underset{\mathrm{L}}{\to}_{\mathsf{d}}^{=} s^{\mathsf{v}}$*.*
4. CbV internal: $t \underset{\mathrm{L}}{\to}_{\beta_v} s$ *iff* $t^{\mathsf{v}} \underset{\neg\mathrm{L}}{\to}_{\beta_!} \underset{\neg\mathrm{L}}{\to}_{\mathsf{d}}^{=} s^{\mathsf{v}}$*; and* $t \underset{\mathrm{L}}{\to}_{\mathbf{o}} s$ *iff* $t^{\mathsf{v}} \underset{\neg\mathrm{L}}{\to}_{\mathbf{o}} \underset{\neg\mathrm{L}}{\to}_{\mathsf{d}}^{=} s^{\mathsf{v}}$*.*

As a consequence, least-level reduction induces factorization in CbN and CbV $\lambda$-calculi as soon as it does in the bang calculus. And, by Proposition 22, it is a normalizing strategy in CbN and CbV as soon as it is so in the bang calculus.

**Theorem 26 (Factorization and normalization by translation).** *Let* $\Lambda_{\mathcal{O}}^{\mathsf{cbn}} = (\Lambda_{\mathcal{O}}, \to_{\beta} \cup \to_{\mathcal{O}})$ *and* $\Lambda_{\mathcal{O}}^{\mathsf{cbv}} = (\Lambda_{\mathcal{O}}, \to_{\beta_v} \cup \to_{\mathcal{O}})$*.*

1. *If* $\Lambda_{!\mathcal{O}}$ *admits least-level factorization* $\mathtt{Fact}(\underset{\mathrm{L}}{\to}, \underset{\neg\mathrm{L}}{\to})$*, then so do* $\Lambda_{\mathcal{O}}^{\mathsf{cbn}}$ *and* $\Lambda_{\mathcal{O}}^{\mathsf{cbv}}$*.*
2. *If* $\Lambda_{!\mathcal{O}}$ *admits least-level normalization, then so do* $\Lambda_{\mathcal{O}}^{\mathsf{cbn}}$ *and* $\Lambda_{\mathcal{O}}^{\mathsf{cbv}}$*.*

A similar result will hold also when extending the pure calculi with a rule $\mapsto_{\rho}$ other than $\mapsto_{\mathbf{o}}$, as long as the translation preserves $\rho$-redexes, back and forth.

*Remark 27 (Preservation of least-level and of normal forms).* Preservation of normal form and least-level is delicate. For instance, it does not hold with the definition CbV translation $(\cdot)^{\mathsf{v}}$ in [15,30]. There, the translation $t = rs \in \Lambda$ would be $t^{\mathsf{v}} = (\iota\,!(r^{\mathsf{v}}))s^{\mathsf{v}}$ and then Proposition 22 and Proposition 25 would not hold: $\iota\,!(r^{\mathsf{v}})$ is a $\beta_!$-redex in $t^{\mathsf{v}}$ (see Remark 11) and hence $t^{\mathsf{v}}$ would not be normal even though so is $t$, and $\ell\ell(t^{\mathsf{v}}) = 0$ even though $\ell\ell^{\mathrm{CbV}}(t) \neq 0$. This is why we defined two distinct case when defining $(\cdot)^{\mathsf{v}}$ for applications, akin to Bucciarelli *et al.* [7].

### 6 Least-level factorization via bang calculus

We have shown that least-level factorization in a bang calculus $\Lambda_{!\mathcal{O}}$ implies least-level factorization in the corresponding CbN and CbV calculi, via forth-and-back translation. The central question now is *how to prove least-level factorization* for a bang calculus: this section is devoted to that, in the pure and applied cases.

**Overview.** Let us overview our approach by considering $\mathcal{O} = \{\mathbf{o}\}$, and $\to = \to_{\beta_!} \cup \to_{\mathbf{o}}$. Since by definition $\underset{L}{\to} = \underset{L}{\to}_{\beta_!} \cup \underset{L}{\to}_{\mathbf{o}}$ (and $\underset{\neg L}{\to} = \underset{\neg L}{\to}_{\beta_!} \cup \underset{\neg L}{\to}_{\mathbf{o}}$), Lemma 8 states that we can *decompose* least-level factorization of $\to$ in three modules:

1. prove least-level factorization of $\to_{\beta_!}$, *i.e.* $\to_{\beta_!}^* \subseteq \underset{L}{\to}_{\beta_!}{}^* \cdot \underset{\neg L}{\to}_{\beta_!}$;
2. prove least-level factorization of $\to_{\mathbf{o}}$, *i.e.* $\to_{\mathbf{o}}{}^* \subseteq \underset{L}{\to}_{\mathbf{o}}{}^* \cdot \underset{\neg L}{\to}_{\mathbf{o}}$;
3. prove the two linear swaps of Lemma 8.

Note that, for each of $\underset{L}{\to}_{\beta_!}$ and $\underset{L}{\to}_{\mathbf{o}}$, the least level is defined with respect to the set of *all* redexes $\mathcal{R} = \mathcal{R}_{\beta_!} \cup \mathcal{R}_{\mathbf{o}}$, so as to have $\underset{L}{\to} = \underset{L}{\to}_{\beta_!} \cup \underset{L}{\to}_{\mathbf{o}}$. This approach solves the issue we mentioned in Example 1.

Clearly, Points 2 and 3 depend on the specific rule $\mapsto_{\mathbf{o}}$. However, the beauty of a modular approach is that Point 1 can be established in general: we do not need to know $\mapsto_{\mathbf{o}}$, only the shape of its redexes given by $\mathcal{R}_{\mathbf{o}}$. In Section 6.1 we provide a general result of least-level factorization for $\to_{\beta_!}$ (Theorem 28). In fact, we shall show a bit more: the way of decomposing the study of factorization that we have sketched, can be applied to study least-level factorization of any reduction $\to = \to_{\beta_!} \cup \to_{\rho}$, as long as $\to$ has a good least-level.

Once (1) is established (once and for all), to prove factorization of a reduction $\to_{\beta_!} \cup \to_{\mathbf{o}}$ we are only left with (2) and (3). In Section 6.3 we show that the proof of the two linear swaps can be reduced to a single, simple test, involving only the $\mapsto_{\mathbf{o}}$ step (Proposition 34). In Section 7, we will illustrate how all elements play together on a concrete case, applying them to non-deterministic $\lambda$-calculi.

#### 6.1 Factorization of $\to_{\beta_!}$ in a bang calculus

We show that $\to_{\beta_!}$ *factorizes* via least-level reduction (Theorem 28). This holds for a definition of $\underset{L}{\to}_{\beta_!}$ (as in Section 4) where the set of redexes $\mathcal{R}$ contains $\mathcal{R}_{\beta_!} \cup \mathcal{R}_{\mathcal{O}}$—this generalization has essentially no cost, and allows us to use Theorem 28 as a module in the factorization of larger reductions containing $\to_{\beta_!}$.

We prove factorization via Takahashi's parallel reduction method [32]. We define a reflexive reduction $\underset{\neg L}{\Rightarrow}_{\beta_!}$ (called parallel internal $\beta_!$-reduction) which fulfills the conditions of Property 7, *i.e.* $\underset{\neg L}{\Rightarrow}_{\beta_!}{}^* = \underset{\neg L}{\to}_{\beta_!}{}^*$ and $\underset{\neg L}{\Rightarrow}_{\beta_!} \cdot \underset{L}{\to}_{\beta_!} \subseteq \underset{L}{\to}_{\beta_!}{}^* \cdot \underset{\neg L}{\Rightarrow}_{\beta_!}$.

The tricky point is to prove that $\underset{\neg L}{\Rightarrow}_{\beta_!} \cdot \underset{L}{\to}_{\beta_!} \subseteq \underset{L}{\to}_{\beta_!}{}^* \cdot \underset{\neg L}{\Rightarrow}_{\beta_!}$ We adapt the proof technique in [2]. All details are in [12]. Here we just give the definition of $\underset{\neg L}{\Rightarrow}_{\beta_!}$.

We first introduce $\Rightarrow_{\beta_!:n}$ with $n \in \mathbb{N} \cup \{\infty\}$ (the parallel version of $\to_{\beta_!:n}$), which fires simultaneously a number of $\beta_!$-redexes at level at least $n \in \mathbb{N}$, and $\Rightarrow_{\beta_!:\infty}$ does not reduce any $\beta_!$-redex: $T \Rightarrow_{\beta_!:\infty} S$ implies $T = S$.

$$\frac{}{x \Rightarrow_{\beta_!:\infty} x} \qquad \frac{T \Rightarrow_{\beta_!:n} T'}{\lambda x.T \Rightarrow_{\beta_!:n} \lambda x.T'} \qquad \frac{T \Rightarrow_{\beta_!:m} T' \quad S \Rightarrow_{\beta_!:n} S'}{TS \Rightarrow_{\beta_!:\min\{m,n\}} T'S'} \qquad \frac{T \Rightarrow_{\beta_!:n} T'}{!T \Rightarrow_{\beta_!:n+1} !T'}$$

$$\frac{T \Rightarrow_{\beta_!:n} T' \quad S \Rightarrow_{\beta_!:m} S'}{(\lambda x.T)!S \Rightarrow_{\beta_!:0} T'\{S'/x\}}$$

The *parallel internal $\beta_!$-reduction* $\underset{\neg L}{\Rrightarrow}_{\beta_!}$ is the parallel version of $\underset{\neg L}{\rightarrow}_{\beta_!}$, which fires simultaneously a number of $\beta_!$-redexes that are not at minimal level. Formally,

$$T \underset{\neg L}{\Rrightarrow}_{\beta_!} S \quad \text{if } T \Rrightarrow_{\beta_!:n} S \text{ with } n = \infty \text{ or } n > \ell\ell(T).$$

**Theorem 28 (Least-level factorization of $\rightarrow_{\beta_!}$).** *Let $\rightarrow_\rho$ be the contextual closure of a rule $\mapsto_\rho$, and assume that $\rightarrow = \rightarrow_{\beta_!} \cup \rightarrow_\rho$ has good least-level in $\Lambda_{!\mathcal{O}}$. Then, $T \rightarrow^*_{\beta_!} S$ implies $T \underset{L}{\rightarrow}_{\beta_!}{}^* \cdot \underset{\neg L}{\rightarrow}_{\beta_!}{}^* S$.*

In particular, as $\rightarrow_{\beta_!}$ has a good least-level (Proposition 19) in $\Lambda_!$, we have:

**Corollary 29 (Least-level factorization in the pure bang calculus).** *In the pure bang calculus $(\Lambda_!, \rightarrow_{\beta_!})$, if $T \rightarrow^*_{\beta_!} S$ then $T \underset{L}{\rightarrow}_{\beta_!}{}^* \cdot \underset{\neg L}{\rightarrow}_{\beta_!}{}^* S$.*

*Surface Digression.* According to Definition 15, $\beta_!$-reduction $\rightarrow_{\beta_!:0}$ at level 0 (called *surface reduction* in Simpson [31]) can only fire redexes at level 0, *i.e.*, redexes that are not inside boxes or other operators. It can be equivalently defined as the closure of $\mapsto_{\beta_!}$ under contexts $\mathbf{S}$ defined by $\mathbf{S} ::= \langle\cdot\rangle \mid \lambda x.\mathbf{S} \mid \mathbf{S}T \mid T\mathbf{S}$. Since $\rightarrow_{\beta_!:0} \subseteq \underset{L}{\rightarrow}_{\beta_!}$, from least-level factorization (Corollary 29) and monotonicity (Proposition 19), a new proof of a result already proven by Simpson [31] follows.

**Corollary 30 (Surface factorization in the pure bang calculus).** *In the pure bang calculus $(\Lambda_!, \rightarrow_{\beta_!})$, if $T \rightarrow^*_{\beta_!} S$ then $T \rightarrow^*_{\beta_!:0} \cdot \rightarrow^*_{\beta_!:k} S$ with $k > 0$.*

#### 6.2 Pure calculi and least-level normalization

Least-level factorization of $\rightarrow_{\beta_!}$ implies in particular least-level factorization for $\rightarrow_\beta$ and $\rightarrow_{\beta_v}$. As a consequence, least-level reduction is a normalizing strategy for all three pure calculi: the bang calculus, the CbN, and the CbV $\lambda$-calculi.

*The pure bang calculus.* The least-level reduction $\underset{L}{\rightarrow}_{\beta_!}$ is a *normalizing strategy* for $\rightarrow_{\beta_!}$. Indeed, it satisfies all ingredients in Lemma 4. Since we have least-level factorization (Corollary 29), same normal forms, and *persistence* (Proposition 19), $\underset{L}{\rightarrow}_{\beta_!}$ is a *complete strategy* for $\rightarrow_{\beta_!}$: if $T \rightarrow^*_{\beta_!} S$ and $S$ is $\beta_!$-normal, then $T \underset{L}{\rightarrow}_{\beta_!}{}^* S$.

We already observed (Example 21) that the least-level reduction $\underset{L}{\rightarrow}_{\beta_!}$ is non-deterministic, because several redexes at least level may be available. Such non-determinism is however harmless and inessential, because $\underset{L}{\rightarrow}_{\beta_!}$ is *uniform.*

**Lemma 31 (Quasi-Diamond).** *In the pure bang calculus $(\Lambda_!, \rightarrow_{\beta_!})$, the reduction $\underset{L}{\rightarrow}_{\beta_!}$ is quasi-diamond (Property 5), and therefore uniform.*

Putting all the ingredients together, we have (by Lemma 4):

**Theorem 32 (Least-level normalization).** *In the pure bang calculus $(\Lambda_!, \rightarrow_{\beta_!})$, the least-level reduction $\underset{L}{\rightarrow}_{\beta_!}$ is a normalizing strategy for $\rightarrow_{\beta_!}$.*

Theorem 32 means not only that if $T$ is weakly $\beta_!$-normalizing then $T$ can reach its normal form by just performing least-level steps, but also that performing *whatever* least-level steps eventually leads to the normal form, if any.

*Pure CbV and CbN λ-calculi.* By forth-and-back translation (Theorem 26) the least-level factorization and normalization results for the pure bang calculus immediately transfers to the (pure) CbN and CbV settings.

**Theorem 33 (CbV and CbN least-level normalization).**

– CbN*: In* $(\Lambda, \to_\beta)$, $\underset{L}{\to}_\beta$ *is a normalizing strategy for* $\to_\beta$*.*
– CbV*: In* $(\Lambda, \to_{\beta_v})$, $\underset{L}{\to}_{\beta_v}$ *is a normalizing strategy for* $\to_{\beta_v}$*.*

#### 6.3 Least-level Factorization, Modularly

Least-level factorization of $\to_{\beta_!}$ (Theorem 28) can be used to prove factorization for a more complex calculus. Indeed, a simple and modular *test* establishes least-level factorization of a reduction $\to_{\beta_!} \cup \to_\rho$ ($\to_\rho$ is a reduction added to $\to_{\beta_!}$), by adapting a similar result in [3]. The test relies on the fact that we have already proved Theorem 28, and it *simplifies* Lemma 8: the proof of the two linear swaps of Lemma 8 is reduced to a single, easier check, which only involves the rule $\mapsto_\rho$. As usual, the least level in $\underset{L}{\to}_{\beta_!}$ and $\underset{L}{\to}_\rho$ is defined with respect to the set $\mathcal{R} = \mathcal{R}_{\beta_!} \cup \mathcal{R}_\rho$ of redexes. An example of the use of this test is in Section 7.

**Proposition 34 (Modular test for least-level factorization).** *Let* $\to_\rho$ *be the contextual closure of a rule* $\mapsto_\rho$*, and assume that* $\to = \to_{\beta_!} \cup \to_\rho$ *has a good least-level in* $\Lambda_{!\mathcal{O}}$*. Then* $\to$ *factorizes via* $\underset{L}{\to} = \underset{L}{\to}_{\beta_!} \cup \underset{L}{\to}_\rho$ *if the following hold:*

1. *(*least-level factorization of $\to_\rho$*)* $\to_\rho^* \subseteq \underset{L}{\to}_\rho{}^* \cdot \underset{\neg L}{\to}_\rho{}^*$*;*
2. *(*substitutivity *of* $\mapsto_\rho$*)* $R \mapsto_\rho R'$ *implies* $R\{T/x\} \mapsto_\rho R'\{T/x\}$*;*
3. *(*root linear swap*)* $\underset{\neg L}{\to}_{\beta_!} \cdot \mapsto_\rho \subseteq \mapsto_\rho \cdot \to_{\beta_!}^*$*.*

### 7 Case study: non-deterministic λ-calculi

To show how to use our framework, we apply the tools we have developed on our running example (see Examples 1 and 9). We extend the bang calculus with a *non-deterministic* binary operator $\oplus$, that is, $(\Lambda_{!\oplus}, \to_{\beta_!\oplus})$ where $\to_{\beta_!\oplus} = \to_{\beta_!} \cup \to_\oplus$, and $\to_\oplus$ is the contextual closure of the (non-deterministic) rules:

$$\oplus(T, S) \mapsto_\oplus T \qquad\qquad \oplus(T, S) \mapsto_\oplus S.$$

*First step: non-deterministic bang calculus.* We analyze $\Lambda_{!\oplus}$. We use our modular test to prove least-level factorization for $\Lambda_{!\oplus}$: if $T \to_{\beta_!\oplus}^* U$ then $T \underset{L}{\to}_{\beta_!\oplus}{}^* \cdot \underset{\neg L}{\to}_{\beta_!\oplus}{}^* U$. By Lemma 4, an immediate consequence of the factorization result is that the least-level strategy is *complete*: if $U$ is normal, $T \to_{\beta_!\oplus}^* U$ implies $T \underset{L}{\to}_{\beta\oplus}{}^* U$.

*Second step: CbN and CbV non-deterministic calculi.* By translation, we have *for free*, that the analogous results hold in $\Lambda_\oplus^{\mathsf{cbn}}$ and $\Lambda_\oplus^{\mathsf{cbv}}$, as defined in Example 9. So, least-level factorization holds for both calculi, and moreover

– *CbN completeness*: in $\Lambda_\oplus^{\mathsf{cbn}}$, if $u$ is normal, $t \to_{\beta\oplus}^* u$ implies $t \underset{L}{\to}_{\beta\oplus}{}^* u$.
– *CbV completeness*: in $\Lambda_\oplus^{\mathsf{cbv}}$, if $u$ is normal, $t \to_{\beta_v\oplus}^* u$ implies $t \underset{L}{\to}_{\beta_v\oplus}{}^* u$.

*What do we really need to prove?* The only result we need to prove is least-level factorization of $\to_{\beta_!\oplus}$. Completeness then follows by Lemma 4 and the translations will automatically take care of transferring the results.

To prove factorization of $\to_{\beta_!\oplus}$, most of the work is done, since least-level factorization of $\to_{\beta_!}$ is already established; we then use our test (Proposition 34) to extend $\to_{\beta_!}$ with $\to_{\oplus}$. The only ingredients we need are substitutivity of $\mapsto_{\oplus}$ (which is an obvious property), and the following easy lemma.

**Lemma 35 (Roots).** *Let $\rho \in \{\beta_!, \oplus\}$. If $T \underset{\neg L}{\to}_{\rho} R \mapsto_{\oplus} S$ then $T \mapsto_{\oplus} \cdot \to_{\rho}^{=} S$.*

**Theorem 36 (Least-level factorization in non-deterministic calculi).**

1. *In $(\Lambda_{!\oplus}, \to)$, $\mathtt{Fact}(\underset{L}{\to}, \underset{\neg L}{\to})$ holds for $\to = \to_{\oplus} \cup \to_{\beta_!}$.*
2. *Least-level factorization holds in $(\Lambda_{\oplus}^{\mathtt{cbn}}, \to_{\oplus} \cup \to_{\beta})$, and in $(\Lambda_{\oplus}^{\mathtt{cbv}}, \to_{\oplus} \cup \to_{\beta_v})$.*

*Proof.* 1. It is enough to verify the hypotheses of Proposition 34, via Lemma 35.
2. It follows from Theorem 26 and Theorem 36.1. □

Completeness is the best that can be achieved in these calculi, because of the true non-determinism of $\to_{\oplus}$ and hence of least-level reduction and of any other complete strategy for $\to$. For instance, in $\Lambda_{\oplus}^{\mathtt{cbn}}$ there is no normalizing strategy for $\oplus(x, \delta\delta)$ in the sense of Definition 2, since $x \underset{L}{\leftarrow}_{\oplus} \oplus(x, \delta\delta) \underset{L}{\to}_{\oplus} \delta\delta \underset{L}{\to}_{\beta} \dots$ .

## 8 Conclusions and Related Work

Combining translations (Theorem 26), least-level factorization for $\to_{\beta_!}$ (Theorem 28), and modularity (Proposition 34), gives us a powerful method to analyze factorization in various $\lambda$-calculi that *extend* the pure CbN and CbV calculi. The main novelty is transferring the results from a calculus to another via translations.

*Related Work.* Many calculi inspired by linear logic subsume CbN and CbV, such as [5,6,29,24] (other than the ones already cited). We chose the bang calculus for its simplicity, which eases the analysis of the CbN and CbV translations.

To study CbN and CbV in a uniform way, an approach orthogonal to ours is given by Ronchi della Rocca and Paolini's parametric $\lambda$-calculus [28]. It is a *meta-calculus*, where the reduction rule is *parametric* with respect to a subset of terms (called values) with suitable properties. Different choices for the set of values define different calculi—that is, different reductions. This allows for a uniform presentation of proof arguments, such as the proof of standardization, which is actually a *meta-proof* that can be instantiated in both CbN and CbV.

Least-level reduction is studied for calculi based on linear-logic in [34,1] and for linear logic proof-nets in [8,26]. It is studied for pure CbN $\lambda$-calculus in [2].

*Acknowledgments.* The authors thank Beniamino Accattoli for insightful comments and discussions. This work was partially supported by EPSRC Project EP/R029121/1 *Typed Lambda-Calculi with Sharing and Unsharing*.
